# TuRLK1, a leucine-rich repeat receptor-like kinase, is indispensable for stripe rust resistance of *YrU1* and confers broad resistance to multiple pathogens

**DOI:** 10.1186/s12870-022-03679-6

**Published:** 2022-06-08

**Authors:** Shenghao Zou, Yansheng Tang, Yang Xu, Jiahao Ji, Yuanyuan Lu, Huanming Wang, Qianqian Li, Dingzhong Tang

**Affiliations:** State Key Laboratory of Ecological Control of Fujian-Taiwan Crop Pests, Key Laboratory of Ministry of Education for Genetics, Breeding and Multiple Utilization of Crops, Plant Immunity Center, Fujian Agriculture and Forestry University, Fuzhou, 350002 China

**Keywords:** *Triticum urartu*, *TuRLK1*, Immune response, Stripe rust, Powdery mildew, *YrU1*

## Abstract

**Background:**

YrU1 is a nucleotide-binding site (NBS) and leucine-rich repeat (LRR) protein (NLR), with additional ankyrin-repeat and WRKY domains and confers effective resistance to stripe rust fungus *Puccinia striiformis* f. sp. *Tritici* (*Pst*). YrU1 was positionally cloned in the progenitor species of the A genome of bread wheat, *Tricicum urartu,* recently*.* However, the molecular mechanism and components involved in YrU1-mediated resistance are not clear.

**Results:**

In this study, we found that the transcript level of *TuRLK1*, which encodes a novel leucine-rich repeat receptor-like kinase, was up-regulated after inoculation with *Pst* in the presence of YrU1, through RNA-seq analysis in *T. urartu* accession PI428309. TuRLK1 contained only a small number of LRR motifs, and was localized in the plasma-membrane. Transient expression of *TuRLK1* induced hypersensitive cell death response in *N. benthamiana* leaves. Silencing of *TuRLK1*, using barley stripe mosaic virus (BSMV)-induced gene silencing (VIGS) system in PI428309 that contains YrU1, compromised the resistance against stripe rust caused by *Pst* CY33, indicating that TuRLK1 was required for YrU1-activated plant immunity. Furthermore, overexpression of *TuRLK1* could enhance powdery mildew resistance in bread wheat and *Arabidopsis thaliana* after inoculating with the corresponding pathogens.

**Conclusions:**

Our study indicates that TuRLK1 is required for immune response mediated by the unique NLR protein YrU1, and likely plays an important role in disease resistance to other pathogens.

**Supplementary Information:**

The online version contains supplementary material available at 10.1186/s12870-022-03679-6.

## Background

Plants defend themselves against pathogens with a two-tiered innate immune detection-and-response system, which includes pattern-triggered immunity (PTI) and effector-triggered immunity (ETI) [1]. PTI is activated by recognition of pathogen-/damage-derived molecules via cell surface-localized pattern-recognition receptors (PRRs), whereas ETI is often triggered by perception of pathogen effector proteins via intracellularly localized nucleotide-binding, leucine-rich repeat receptors (NLRs) [2, 3]. PTI and ETI are initiated by distinct activation mechanisms and involve different early signaling cascades, whereas they are not mutually isolated but with intricate interplays [4]. For instance, recent studies have reported that TIR (N-terminal Toll/interleukin-1 domain of NLRs) signaling plays a key role in PTI, and TIR signaling mutants exhibit attenuated PTI responses and decreased resistance against *Pto* DC3000 *hrcC* and *Hyaloperonospora arabidopsidis* (*Hpa*) Noco2 in *Arabidopsis thaliana* (hereafter, *Arabidopsis*) [5]. PTI induced by *Pto* DC3000 *hrcC*, flg22 and nlp20 in *Arabidopsis* requires components of ETI, such as signaling-competent dimers of the lipase-like proteins EDS1 and PAD4, ADR1 family helper NLRs [5, 6]. Moreover, in *Arabidopsis*, ETI activated by NLRs could increase the production of ROS and the expression of PTI-responsive genes induced by PAMPs to enhance PTI defense responses [7]. Nevertheless, on the other hand, potentiation of PTI is an indispensable component of ETI during bacterial infection in *Arabidopsis*, supported by substantial evidence [7, 8]. For example, PRR and its co-receptors *Arabidopsis* mutants, *fls2 efr cerk1*and *bak1 bkk1 cerk1*, could not show an effective ETI response against *Pst* DC3000 (*avrRpt2*, *AvrPphB* or *AvrRps4*) [8]. Similarly, two other mutants, *bak1-5 bkk1-1* and *fls2 efr*, both showed a higher susceptibility to *Pst* DC3000 (*AvrRps4*) than wild-type plants [7]. Notably, PTI was demonstrated being able to potentiate ETI-induced cell death. Activation of PTI or ETI^AvrRpp4/AvrRps4^ alone could not result in cell death, except for co-activation of PTI and ETI^AvrRpp4/AvrRps4^. Those findings indicate that ETI and PTI mutually potentiate and interdepend with each other. However, those studies are performed only in model plants *Arabidopsis* with bacterial pathogens.

PRRs mainly consist of receptor-like kinases (RLKs) and receptor-like proteins (RLPs). In plants, RLKs are a large family of proteins, which contain an ectodomain (ECD), a single pass transmembrane domain, and a cytoplasmic kinase domain, while RLPs lack the intracellular kinase domain [9, 10]. The ECDs of RLKs are highly variable, including leucine-rich repeat (LRR) domain, lysine motifs (LysM), lectin domain, or epidermal growth factor (EGF)-like domain, providing means to recognize a wide range of ligands, such as steroids, peptides, polysaccharides, and lipopolysaccharides [9]. Notably, members of the LRR-RLK subfamily contain varied numbers of LRRs, and the role of the LRR-RLK is often dependent on the number of LRRs in the PTI. In *Arabidopsis*, LRR-RLKs with large numbers of LRRs often function as PRRs to perceive PAMPs or danger signals, such as FLS2, EFR, and PEPR1/2, which contain more than 20 LRRs. In contrast, LRR-RLK proteins with small numbers of LRRs often function as co-receptors for PRRs, such as BAK1 and SOBIR1, or regulatory proteins in the immune complex, such as BIR1 and BIR2, which contain only 4 to 6 LRRs.

Stripe rust, caused by biotrophic pathogen *Pst*, is one of the most devastating diseases of wheat (*Triticum aestivum* L.) and severely reduces bread wheat yields around the world [11–13]. To date, although over 80 stripe rust resistance loci have been identified and mapped in *Triticum spp*., only nine genes, *Yr5*, *Yr7*, *YrSP*, *Yr15*, *Yr18/Lr34*, *Yr36*, *Yr46*, *YrAS2388* and *YrU1*, have been cloned [14–22]. Among them, *YrAS2388* encodes a typical coiled-coil (CC) NLR, and *Yr5*, *Yr7* and *YrSP* all encode NLRs with a non-canonical N-terminal zinc-finger BED domain [19]. *YrU1* encodes an NLR with an N-terminal ankyrin-repeat (ANK) domain and a C-terminal WRKY domain, which was cloned in *Triticum urartu*, the progenitor species of the A genome of bread wheat. The NLRs with ANK and WRKY domains are rare, and only exist in the genomes of wheat and its relatives [15]. YrU1 likely functions as a typical NLR that elicits effective ETI after recognition of the cognate effector proteins derived from biotrophic pathogen *Pst*. How YrU1 activates plant immunity, and whether pattern-recognition receptors/co-receptor or other key components of PTI are required for YrU1-mediated plant immunity are remained to be determined.

In the RNA-seq data of *T. urartu* accession PI428309, which contains the functional *YrU1* gene, we found the expression of a novel leucine-rich repeat receptor-like kinase gene, designated *TuRLK1*, was induced after infection of *Pst* CYR33. *TuRLK1* contains six LRR motifs, localizes in the plasma-membrane. Overexpression of *TuRLK1* could induce strong cell death in *N. benthamiana* leaves, suggesting that *TuRLK1* may play an important role in plant immune response. Silencing of *TuRLK1*, using barley stripe mosaic virus (BSMV)-induced gene silencing (VIGS) system in PI428309, severely compromised the stripe rust resistance of *YrU1* against *Pst* CY33, indicating that TuRLK1 was required for YrU1-mediated plant immunity. This study provides evidence that an RLK, the key PTI component, is indispensable for ETI, in the fungal disease resistance, which is in consistent with the previous studies on bacterial disease resistance. Furthermore, over-expression of *TuRLK1* could enhance the powdery mildew resistance in bread wheat cultivar Fielder and *Arabidopsis thaliana* accession Col-0, which indicates the potential role of *TuRLK1* in resistance breeding against multiple disease pathogens.

## Result

### The expression of *TuRLK1* was up-regulated after inoculation with *Pst* CY33 in *Triticum urartu* PI428309

**Fig. 1:**
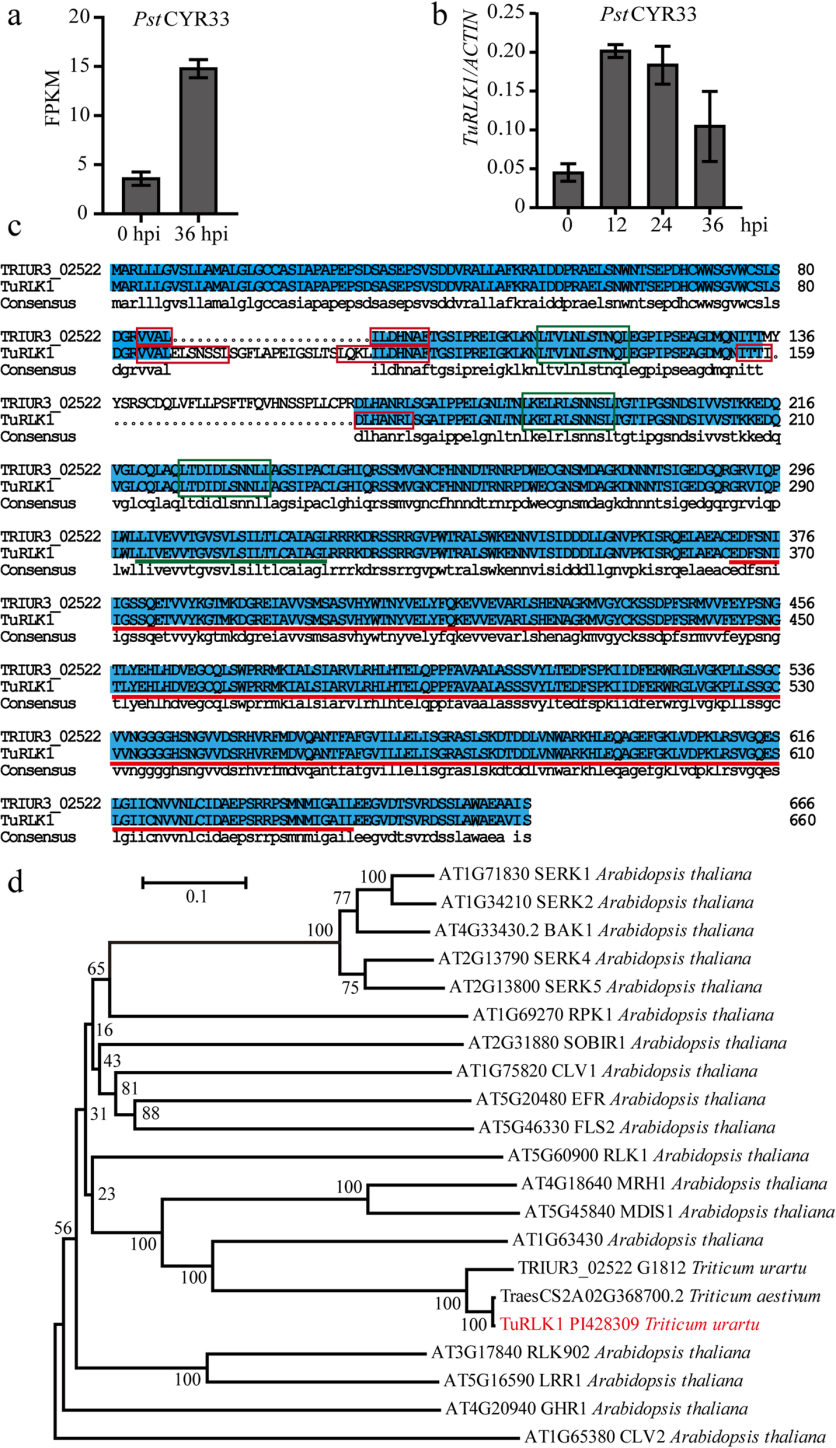
Characterization of *TuRLK1* in PI428309 and G1812. **a** RNA-seq data showed that the transcript level of *TuRLK1* in PI428309 was significantly induced after *Pst* CY33 infection. FPKM: Fragments Per Kilobase of exon model per Million mapped fragments. hpi: hours post inoculation. **b** Transcript levels of *TuRLK1* in PI428309 were examined by qRT-PCR, using *ACTIN* as an internal control. Leaves at the seedling stage were detached for RNA isolation and qRT-PCR after inoculation with *Pst* CY33 at the indicated time points. Error bars represent the standard deviation of 3 independent biological replicates. **c** Protein sequences alignment between TuRLK1 in PI428309 and G1812. The protein sequences shaded in blue are identical. Domains in the protein sequences were predicted according to the SMART program. LRR motifs are in the red or blue rectangular blocks. Red line indicates the kinase domain, and green line highlights the transmembrane region. **d** Phylogenetic tree analysis with TuRLK1 and 20 other RLKs. The phylogenetic tree, constructed according to the protein sequences of the 21 RLKs under the neighbor-joining method, was depicted using MEGA5.0 software

In order to identify important PTI components involved in *YrU1*-mediated stripe resistance, RNA-seq data was analyzed in *T. urartu* accession PI428309 [23], which has the functional *YrU1* gene. The transcripts of a novel leucine-rich repeat receptor-like kinase gene, designated *TuRLK1*, were increased significantly after *Pst* CYR33 infection in PI428309 (Fig. 1a)*.* We verified the results of RNA-seq via examining the transcript levels of *TuRLK1* at 0 h post inoculation (hpi), 12 hpi, 24 hpi and 36 hpi with *Pst* CYR33 by qRT-PCR. The accumulation of transcripts of *TuRLK1* was at much higher level at 12 hpi, 24 hpi and 36 hpi as shown in Fig. 1b. These results implied that *TuRLK1* may be involved in the resistance to wheat stripe rust conferred by *YrU1*.

The coding sequence of *TuRLK1* in PI428309 encodes a protein of 660 amino acids, and the relative molecular weight of TuRLK1 is about 73 kDa. TuRLK1, with an N-terminal LRR domain, a transmembrane region and a C-terminal Ser/Thr/Tyr Kinase domain, has the typical structure of RLK family members (Fig. 1c). TRIUR3_02522, the TuRLK1 in the susceptible *T. urartu* accession G1812, has 666 amino acids, which is nearly identical to TuRLK1 in PI428309, except for the number of LRR motifs. TuRLK1 in PI428309 has six LRR motifs, with two more LRR motifs than TRIUR3_02522 (Fig. 1c). As shown in Fig. 1d, the protein sequence of TuRLK1 in PI428309 is greatly different from the well-known RLKs in *Arabidopsis thaliana*. To identify the allelic variants of TuRLK1 in the Triticeae, we searched protein databases and conducted a multiple sequence alignment with TuRLK1 and 4 homologous proteins of TuRLK1 from different Triticeae accessions. The alleles identified from different Triticeae accessions are almost identical, except for a few amino acids (Additional file 1), indicating that TuRLK1 is well conserved in the Triticeae.

### TuRLK1 is localized in plasma membrane

**Fig. 2:**
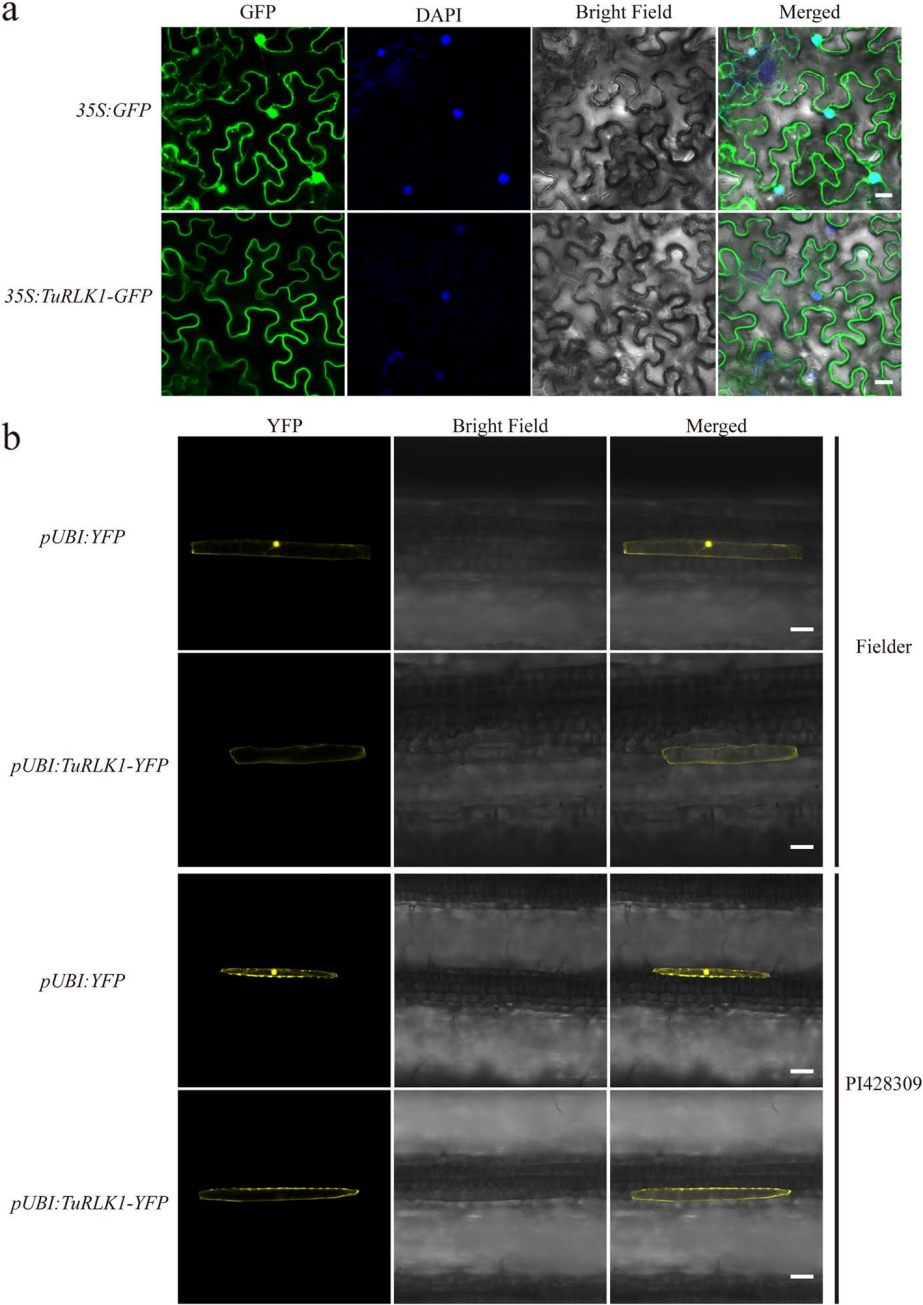
Subcellular localization of TuRLK1 in *N. benthamiana*, bread wheat and *Triticum urartu*. **a** TuRLK1 mainly localized in the plasma-membrane of *N. benthamiana* cells. *TuRLK1* fused *GFP* report gene in the C-terminus was driven by 35S promoter. The localization of TuRLK1 was observed using confocal microscope, 48 h after infiltration with *Agrobacterium* strain GV3101. DAPI (4’, 6-diamidino-2-phenylindole) staining of the nuclear compartment as control. Bar = 20 μm. **b** TuRLK1 localized in the plasma-membrane of bread wheat cultivar Fielder and *Triticum urartu* PI428309 cells. *TuRLK1* fused *YFP* report gene in the C-terminus was driven by ubiquitin promoter. The localization of TuRLK1 was observed 36 h after single-cell transient expression on plant leaves. Bar = 50 μm

For examining the localization of TuRLK1, we created two constructs expressing TuRLK1 and Green/Yellow Fluorescent Protein (GFP/YFP) fusion proteins, respectively. After transiently expressing them in *Nicotiana benthamiana* and wheat leaves, TuRLK1 proteins were observed mainly localized in plasma membrane of both *N. benthamiana* and wheat cells using confocal microscope (Fig. 2a and b). This result is consistent with the localization characteristics of most reported RLKs.

### Overexpression of *TuRLK1* could induce cell death in *N. benthamiana* leaves

**Fig. 3:**
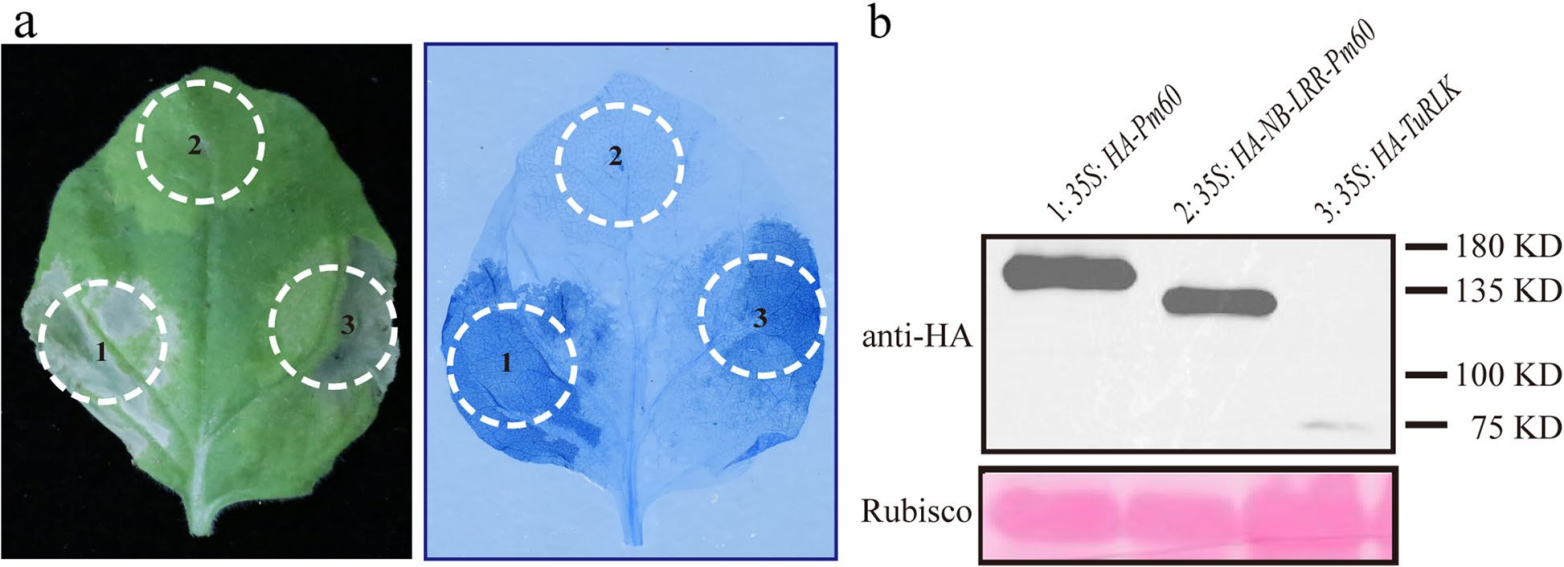
Overexpression of *TuRLK* induced hypersensitive cell death response in *N. benthamiana* leaves. **a** *35S: HA-TuRLK* was transiently expressed in *N. benthamiana*. *35S: HA-Pm60* was used as positive control to induce HR, whereas *35S: HA-NB-LRR-Pm60* as a negative control. The infiltrated leaves were photographed (left panel) and then stained with trypan blue (right panel) to visualize HR at 2 days post inoculation (dpi). **b** Accumulation of proteins was examined by immunoblot analysis

To gain more insight into the function of TuRLK1, we transiently overexpressed TuRLK1 in *N. benthamiana* leaves. As shown in Fig. 3a, overexpression of the full-length TuRLK1 in *N. benthamiana* leaves induced hypersensitive cell death response (HR) in the injection region; however, the cell death phenotype was not as strong as that of the Pm60 protein, which is an NLR protein in PI428309, a control used [24]. By contrast, the NB-LRR domain of Pm60, which lacks the coiled-coil domain, did not induce cell death consistent with the previous reports (Fig. 3a). Additionally, HA-TuRLK1 protein was correctly expressed in *N. benthamiana* tested by immunoblot analysis (Fig. 3b).

### Silencing of *TuRLK1* in PI428309 compromised the resistance of *YrU1* against *Pst* CY33

**Fig. 4:**
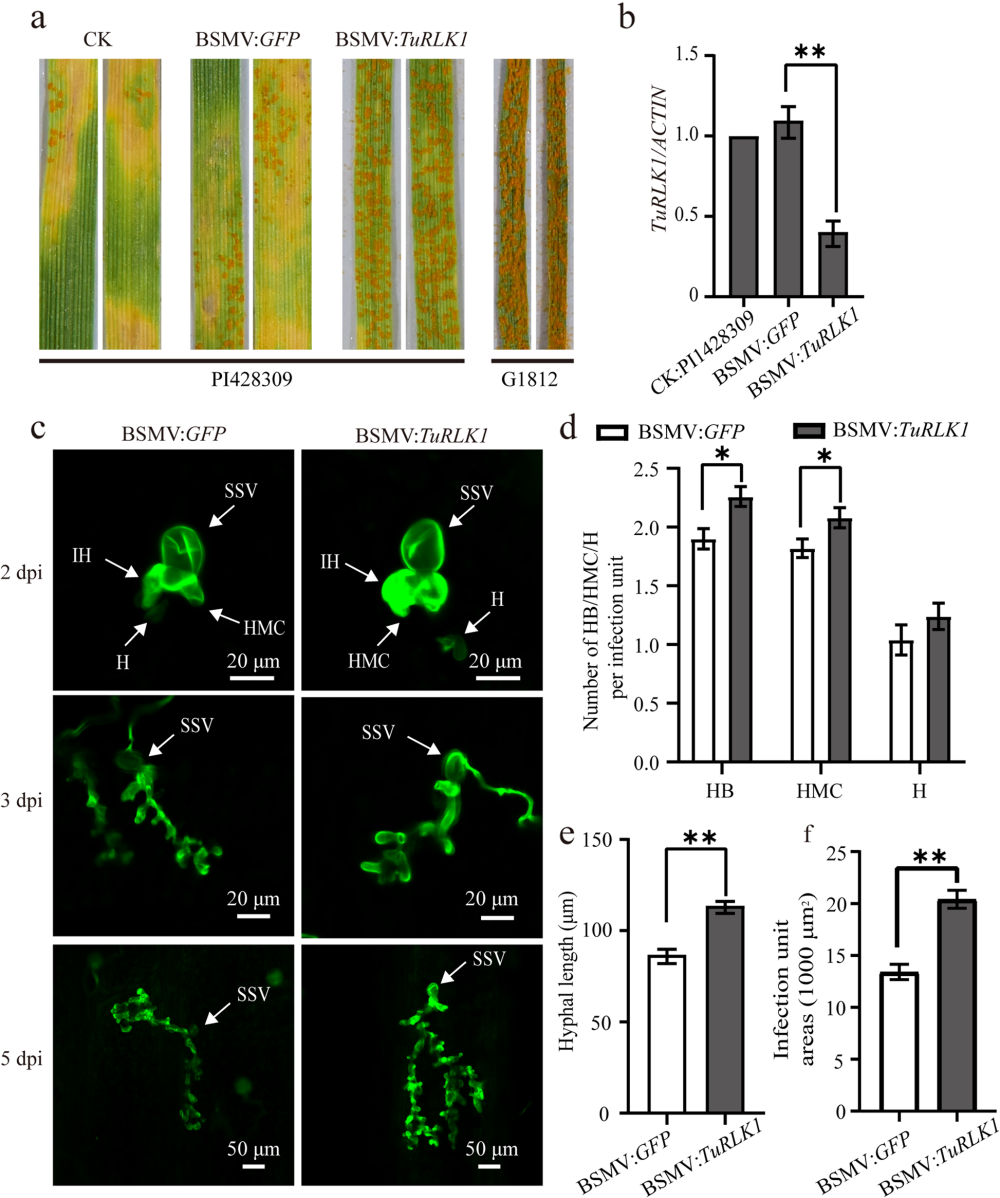
BSMV-induced silencing of *TuRLK1* compromised the resistance of YrU1 to *Pst* CYR33 in PI428309. **a** PI428309 plants were infected with BSMV on the second leaf and inoculated with urediniospores of *Pst* CYR33 on the fourth leaf 21 days after infection. Photographs were taken at 14 dpi. BSMV: *TuRLK1*: Barley stripe mosaic virus-induced *TuRLK1* silencing in PI428309; BSMV: *GFP*: Barley stripe mosaic virus-induced *GFP* silencing in PI428309, as control; G1812: G1812, without BSMV infection, as control; CK PI428309: PI428309, without BSMV infection, as control. **b** The expression of *TuRLK1* was examined by quantitative reverse transcription PCR (qRT-PCR) before inoculation with *Pst* CYR33. *ACTIN* was used as an internal control. Error bars represent ± SD of values obtained from at least three independent biological samples. Statistically significant difference (Student’s t-test): **, *P* < 0.01. **c** The morphology of mycelium of *Pst* CYR33 was observed using WGA staining and histological analysis at 2 dpi, 3 hpi and 5 hpi. Bars are shown in the corresponding picture. SSV: substomatal vesicles; IH: infection hyphae; HMC: haustoria mother cell; H: haustoria. **d** The numbers of HB, HMC and H of *Pst* were counted and analyzed at 2 dpi. HB: hyphal branches. **e** The length of IH was measured in each infected site at 3 dpi. **f** The extended infection area was measured at 5 dpi. Error bars represent ± SE of values obtained from three independent experiments (*n* = 50) in (d), (e), (f). Statistically significant difference (Student’s *t*-test): *, *P* < 0.05; **, *P* < 0.01

To study whether *TuRLK1* is involved in the *YrU1*-conferred stripe rust resistance, we knockdown *TuRLK1* via BSMV-induced gene silencing (VIGS) system in PI428309, the *T. urartu* accession that contains the functional *YrU1*. At 14 days post inoculation, the uredia were denser on the leaves with knockdown of *TuRLK1* contrasting to those on the control leaves; nearly no necrotic spot caused by HR was observed, displaying more susceptible symptoms (Fig. 4a, b). Thus, silencing of *TuRLK1* compromised the resistance of *YrU1* to stripe rust *Pst* CYR33, and *TuRLK1* is indispensable for stripe rust resistance conferred by *YrU1*.

To further characterize the stripe resistance on the leaves with reduced *TuRLK1* expression, the infected leaves of PI428309 were collected at 2 days post inoculation (2 dpi), 3 dpi and 5 dpi, and wheat germ agglutinin (WGA) staining was used for visualizing the mycelium of *Pst*, which was observed by confocal microscope. We counted the number of hyphal branches (HB), haustorial mother cells (HMC) and haustoria (H) at 2 dpi, and the length of infection hyphae and the infection unit area were measured at 3 or 5 dpi, respectively. As shown in Fig. 4d, significant differences were existing in the number of HB, HMC except for H, between the *TuRLK1* knockdown plants and the control samples (Fig. 4c). Similarly, in *TuRLK1* silenced leaves, the length of infection hyphae and the infection unit area were significantly larger than those in the control leaves (Fig. 4e, f). Taken all together, these results showed that *TuRLK1* contributes to *YrU1*-mediated stripe rust resistance.

### The wheat powdery mildew resistance was potentiated after transient overexpression of *TuRLK1*

**Fig. 5:**
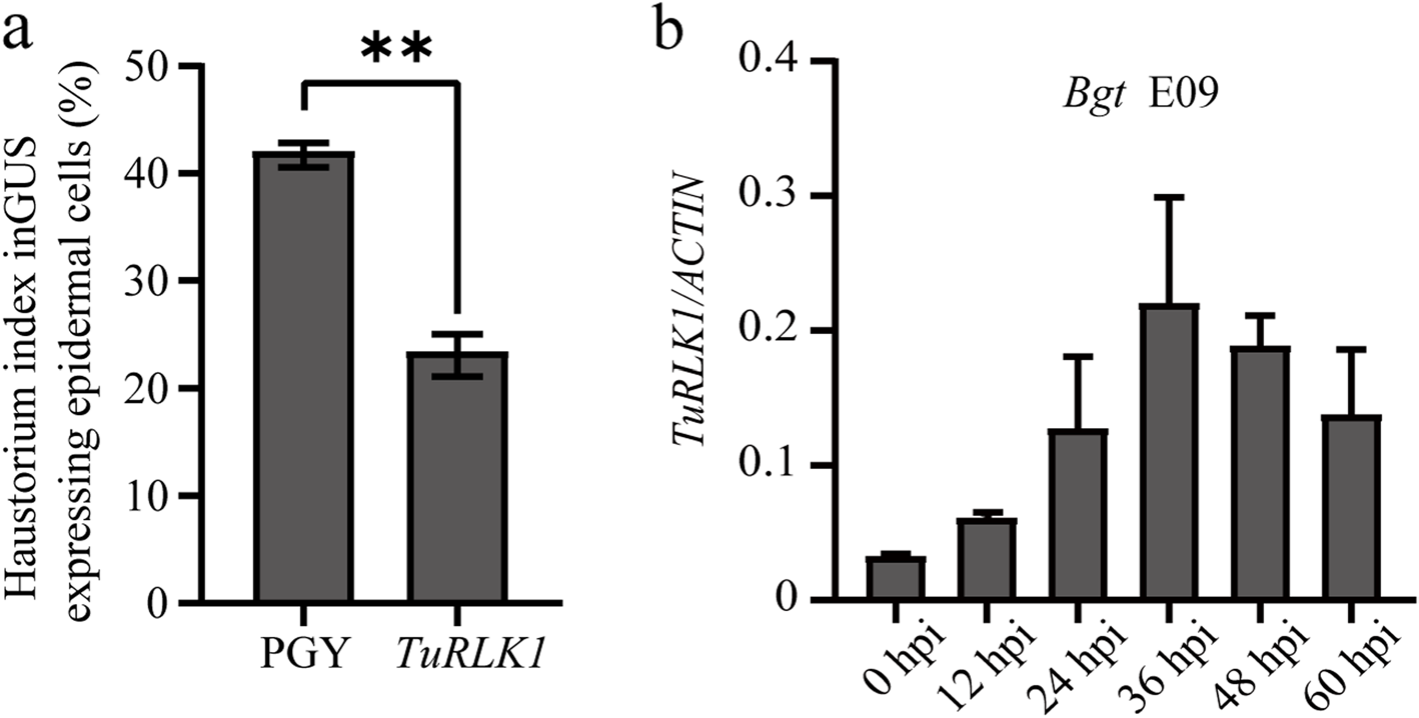
Transient overexpression of *TuRLK1* increased the powdery mildew resistance in common wheat. **a** Single-cell transient overexpression of *TuRLK1* on the detached leaves of wheat cultivar Fielder significantly decreased the haustorium index after inoculation with *Bgt* E09, in contrast to the control *PGY*. Error bars represent ± SD of values obtained from three independent experiments. Statistically significant differences (Student’s t-test): **, *P* < 0.01. **b** Transcript levels of *TuRLK1* were examined in PI428309 by qRT-PCR, using *ACTIN* as an internal control. Leaves at the seedling stage were detached after inoculation with *Bgt* E09 at the indicated time points for qRT-PCR. Error bars represent the standard deviation of 3 independent biological replicates

To investigate whether *TuRLK1* is also involved in basal defense or disease resistance to other pathogens besides *YrU1*-mediated stripe rust resistance, we assessed the role of *TuRLK1* on the wheat powdery mildew resistance using single-cell transient gene expression system mediated by particle bombardment. In this assay, we transiently expressed *TuRLK1* in the susceptible bread wheat cultivar Fielder, and then inoculated the leaves with powdery mildew pathogen *Blumeria graminis* f. sp. *tritici* (*Bgt*) E09. As shown in Fig. 5a, the haustorium index in the leaves of Fielder was significantly decreased when transiently expressing *TuRLK1* comparing to expression of PGY, a control used, which indicating that *TuRLK1* plays an important role in resistance against *Bgt* E09. In addition, we examined the transcript levels of *TuRLK1* in PI428309 by qRT-PCR at 0 hpi, 12 hpi, 24 hpi, 36 hpi, 48 hpi and 60 hpi, after *Bgt* E09 infection. As shown in Fig. 5b, the expression of *TuRLK1* increased significantly after infection, which suggesting that *TuRLK1* may play a role in the resistance to wheat powdery mildew too.

### TuRLK1 enhanced the resistance to powdery mildew in *Arabidopsis thaliana*

**Fig. 6:**
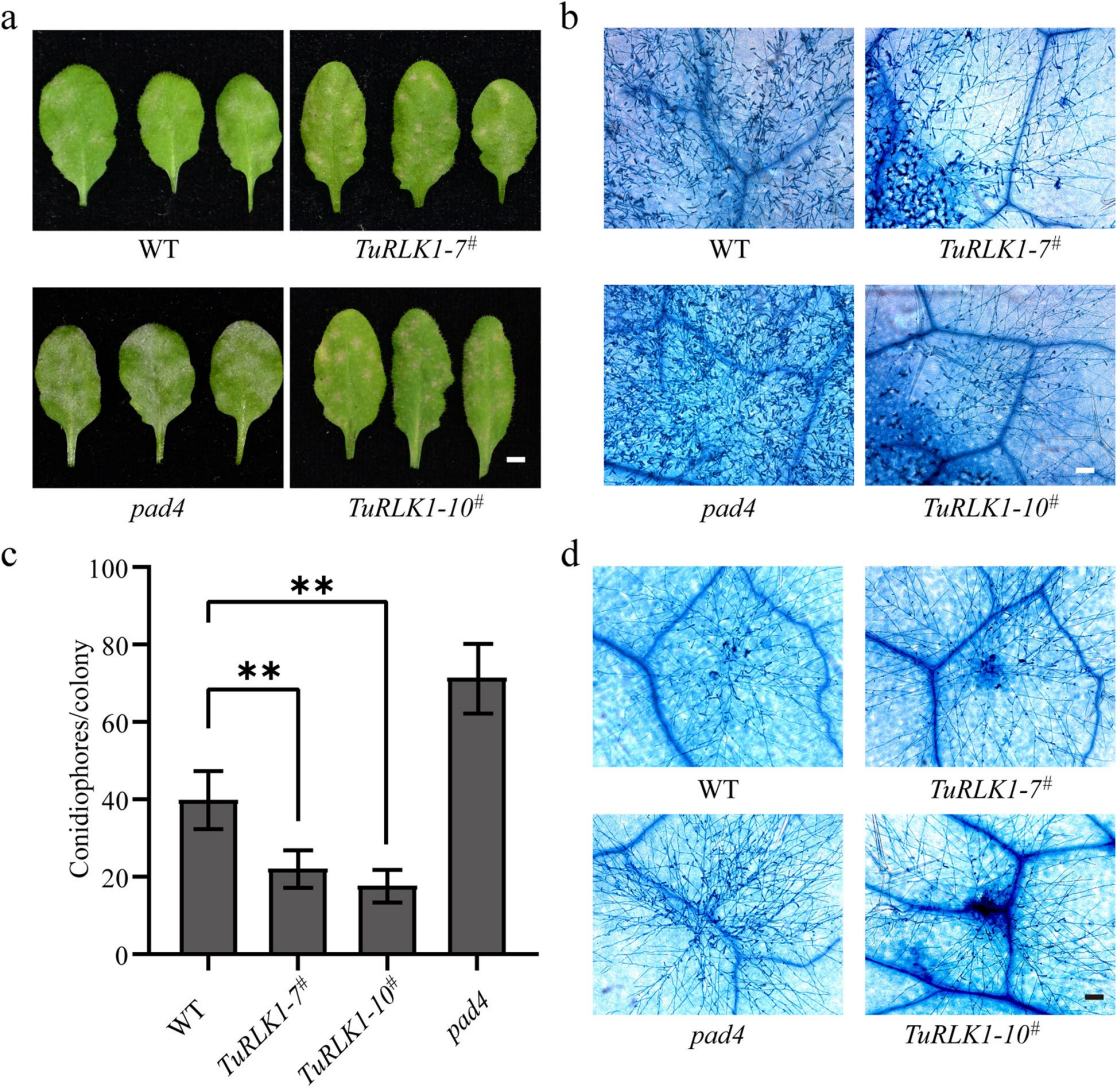
Overexpression of *TuRLK1* enhanced resistance to *G. cichoracearum* in *Arabidopsis.* **a** Four-week-old plants were inoculated with *G. cichoracearum* and photographs were taken at 7 dpi for the representative leaves. WT: *Arabidopsis thaliana* accession Col-0; *pad4*: *Arabidopsis* mutant, as negative control; *TuRLK1-7*^*#*^, *TuRLK1-10*^*#*^: two *TuRLK1* transgenic lines of T_2_ generation. Bar = 8 mm. **b** The leaves shown in (a) were stained with Trypan blue to visualize fungal structures and plant cell death. Bar = 100 μm. **c** Conidiophore formation was assessed in WT, *pad4*, *TuRLK1-7*^*#*^ and *TuRLK1-10*.^*#*^ plants at 5 dpi. Error bars represent ± SE of values obtained from three independent experiments (*n* > 30). Statistically significant difference (Student’s *t*-test): **, *P* < 0.01. **d** Trypan blue staining of the leaves for quantitative analysis of conidiophore formation in (c) after inoculation with *G. cichoracearum* at 5 dpi. Bar = 100 μm

To further investigate the function of *TuRLK1* in disease resistance, we overexpressed *TuRLK1* in *Arabidopsis thaliana*, and then assessed the powdery mildew resistance of the transgenic plants. In this experiment, *TuRLK1* was fused with a HA tag mediated by 35S promoter, then, transformed into *Arabidopsis thaliana* accession Col-0. We inoculated two independent transgenic lines overexpressing *TuRLK1* (*TuRLK1-7*^*#*^, *TuRLK1-10*^*#*^) with fungus *G. cichoracearum*, which is the powdery mildew pathogen of *Arabidopsis thaliana*. The leaves of the two representing overexpressed lines showed much weaker growth of powdery mildew fungi in contrast to the control samples, and displayed some necrotic spots caused by cell death at 7 dpi (Fig. 6a, b). To quantify the fungal growth, we counted the number of conidiophores at 5 dpi. As shown in Fig. 6c, d, the conidiophores per colony in overexpressed plants were significantly less than that in control plants. Taken together, these results indicate that overexpression of *TuRLK1* in *Arabidopsis thaliana* could enhance the resistance against *G. cichoracearum* and cause mild cell death in the leaves of transgenic plants.

## Discussion

Many LRR-RLKs act as PRRs or PRRs co-receptors and play key roles in plant immunity [25]. In this study, we showed that TuRKL1 is involved in stripe rust resistance mediated by YrU1. TuRLK1 belongs to the LRR-RLK subfamily and contains only six LRRs in PI428309. Therefore, it is unlikely that TuRLK1 functions as a PRR, and it may rather function as a PRRs co-receptor or as a signaling protein in the immune complex that assists transmit the immune signal in PTI. Intriguingly, compared with the TuRLK1 in the *T. urartu* susceptible accession G1812, TuRLK1 in PI428309 has two additional LRR motifs in its ectodomain (Fig. 1c). It would be very interesting to investigate whether those TuRLK1 variants have distinct immune function. Previously, we showed that the transgenic plants of YrU1 in Bobwhite background are able to confer stripe rust resistance [15]. It is likely that Bobwhite has a homologous gene of TuRLK1 with a similar function. Consistent with this notion, a highly similar protein of TuRKL1 was identified in Chinese Spring (Additional file 1).

It is interesting that TuRLK1, a PTI component, is required for ETI activated by NLR YrU1 after *Pst* CY33 infection. It is consistent with recent finding that ETI co-opts part of the PTI machinery as an indispensable component [8]. Nevertheless, how TuRLK1 functions in YrU1-mediated ETI is unclear. Besides TuRLK1, what other key immune components are required for the ETI triggered by YrU1 is also unknown. The recent studies in *Arabidopsis* indicated that a robust level of BIK1 and RBOHD, which mediates ETI^ROS^ generation, full immunity-associated gene expression and disease resistance during ETI, is essential for functioning synergistically between the two primary classes of plant immune receptors, PRRs and NLRs [7, 8]. Although, this conclusion was based on research regarding to bacterial disease and in *Arabidopsis*. However, it would be interesting to examine whether BIK1 and RBOHD are also the key immune components that contribute to the stripe rust resistance of YrU1 in wheat.

In addition to playing an important role in YrU1-mediated stripe rust resistance, TaRLK1 appears to confer broad resistance to other pathogens, as transient overexpression of TuRLK1 decreased the haustorium index and enhanced the resistance to powdery mildew in wheat. Furthermore, stable overexpression of TuRLK1 could also enhance the resistance against powdery mildew in *Arabidopsis*. One possibility is that TuRLK1 may act as a co-receptor or signaling protein that potentiates PTI. In this scenario, overexpression of *TuRLK1* in wheat and *Arabidopsis thaliana* may enhance the function of associated PRR and result in production of reactive oxygen species (ROS), activation of mitogen-activated protein kinases (MAPKs) and induction of defense genes.

Although previously we showed that a NAC transcription factor TuNAC69 contributes to YrU1-mediated resistance in *T. urartu* [23], the components required for YrU1-mediated resistance are largely unknown. In this study, we identified an RLK, TuRLK1 that functions in plant immunity. TuRLK1 localizes in the plasma membrane and its expression is induced in *Triticum urartu* PI428309 after inoculation with *Pst*. We demonstrated that TuRLK1 is indispensable for the stripe rust resistance mediated by YrU1, an NLR with atypical domains, and TaRLK1 also enhances resistance to powdery mildew after overexpression in wheat and *Arabidopsis thaliana*. The results shed light on the understanding of the mechanism of immune response mediated by YrU1. Moreover, this study provides new evidence that PTI components are required for NLR-mediated plant immunity, to the fungal disease resistance in wheat, besides *Arabidopsis* model plants. However, some important questions are remained to be addressed, including how TuRLK1-associated PRR immune complex recognizes the pathogens and transduces the immune signal; what are the PRRs interacting with TuRLK1, and whether other key PTI components are required for YrU1-mediated stripe rust resistance.

## Conclusion

Our study found that the expression of a leucine-rich repeat receptor-like kinase TuRLK1 was up-regulated after *Pst* CYR33 infection. TuRLK1 was plasma-membrane localized and could induce hypersensitive cell death response in *N. benthamiana* leaves. Transiently silencing *TuRLK1* in diploid wheat *Triticum urartu* PI428309 that contains YrU1 by VIGS could compromise the resistance to stripe rust. Furthermore, overexpression of *TuRLK1* in common wheat and *Arabidopsis* enhanced the resistance against powdery mildew. In summary, our work indicates that TuRLK1 is required for immune response to stripe rust mediated by NLR protein YrU1, and may also play an important role in disease resistance to other pathogens. This study provides new insight into the role of YrU1 and TuRLK1 in disease resistance, and may reveal potential connections between ETI and PTI in fungal disease.

## Methods

### Plant materials and growth conditions

The *Triticum urartu* accession PI428309, containing the stripe rust resistance gene *YrU1*, originated from El Beqaa, Lebanon, whereas the susceptible accession G1812 originated from Mardin, Turkey [24]. The winter bread wheat cultivar Kn199, which is highly susceptible to powdery mildew, was used to maintain the strain *Bgt* E09 for executing inoculation experiments. The winter bread wheat cultivar Mingxian169 was used to maintain the *Pst* CYR33 for the following assays. The foregoing materials were vernalized in preparation for sowing. The spring bread wheat cultivar Fielder is highly susceptible to *Bgt* E09, which is used in single-cell transient gene expression assay and maintaining *Bgt* E09. For phenotyping, these *Triticum spp*. plants were grown in the glasshouse at 20–22 °C under a 12-h: 12-h, light: dark photoperiod with about 60% relative humidity. *T. urartu* accession PI428309 and bread wheat cultivar Mingxian169, being inoculated with *Pst* CYR33, was cultured in the greenhouse at 16 °C under a 14-h: 10-h, light: dark photoperiod with about 80% relative humidity. Bread wheat cultivar Fielder inoculated with *Bgt* E09 was grown in an incubator at 22 °C under a 12-h-light:12-h- dark photoperiod with about 70% relative humidity [23]. *Arabidopsis thaliana* and *Nicotiana benthamiana* plants were cultured in a growth room at 20–22 °C with a 9-h-light/15-h-dark cycle for phenotyping and a 16-h-light/8-h-dark cycle for seed setting, under light intensity of 7,000–8,000 lx [26, 27]. Powdery mildew strain *Golovinomyces cichoracearum* UCSC1 was maintained with *Arabidopsis* mutant *pad4*, which was grown in an incubator after inoculation under the same conditions as infected Fielder [28].

### Pathogen infection

Inoculation of *Pst* CYR33 was performed as described previously [15, 29, 30]. During the inoculation, talcum powder was mixed with urediospores as an indicator on the leaves. After inoculation, plants were brought to an incubator at 10 °C with 100% relative humidity in the dark for 24 h, immediately. Then, the plants were moved to a greenhouse under the conditions mentioned above. The stripe rust infection type (IT) was evaluated at 14 dpi, basing on a 0–4 scale: 0, immune, no visible uredia and necrosis on leaves; 0;, nearly immune, no uredia with hypersensitive flecks on leaves; 1, very resistant, few small uredia with distinct necrosis on leaves; 2, moderately resistant, few small- to medium sized uredia with dead or chlorosis on leaves; 3, moderately susceptible, a lot of medium-sized uredia, no necrosis, but with chlorosis on leaves; 4, highest susceptible, a large number of large-sized uredia without necrosis on leaves [15]. Plants with IT = 3–4 were susceptible. Inoculations of *Bgt* E09 and *Golovinomyces cichoracearum* UCSC1 were also performed according to previous publications [24, 27, 31].

### RNA isolation, RNA-sequencing and assembling the reads

At the two-leaf stage, the first leaves of PI428309 were detached to extract RNA using the RNeasy Plant Mini Kit (cat. no./ID:74,903; Qiagen). An Agilent 2100 was employed to check the quality of RNA (Agilent Technologies Inc., Santa Clara, CA, USA). RNA-Seq was conducted as described previously [32–34], conducting by the Beijing Genomics Institute (BGI) using the BGISEQ-500 platform. All the reads were cleaned and assembled using the CLC GENOMIC WORKBENCH software (https://www.qiagenbioinformatics.com/products/clc-genomics-workbench/).

### Quantitative reverse transcription PCR (qRT-PCR)

Total RNA extraction and qRT-PCR were performed as previous publication [35]. M-MLV Reverse Transcriptase was used to reverse-transcribe total RNA (Promega, Madison, WI, USA). Transcript levels were examined by qRT-PCR on a BIO-RAD CFX Connect Real-Time System (Bio-Rad Laboratories, Hercules, CA, USA). *ACTIN* was used as the internal control and Student’s *t*-test was performed to evaluate quantitative variation.

### Subcellular localization assay in *Nicotiana benthamiana*

*Agrobacterium* strain GV3101 carrying the recombinant plasmid *35S:TuRLK1-GFP* and P19 were suspended to OD600 = 1.5 with infiltration buffer (10 mM MES, 10 mM MgCl2, 120 µM Acetosyringone) and co-expressed in *N. benthamiana* leaves as described previously [27]. GFP signals were visualized using confocal microscope, 48 h after infiltration.

### Staining and microscopy

In order to visualize cell death in *N. benthamiana* leaves, the infiltrated leaves were stained with trypan blue solution, boiled for 10 min, and decolorized overnight. Then, the cell death in *N. benthamiana* leaves after staining was directly photographed, using a digital single lens reflex camera [36].

### Immunoblot analysis

*Agrobacterium* strain GV3101 carrying the relevant plasmids were suspended to OD_600_ = 1.5 with infiltration buffer [37]. *N. benthamiana* leaves were ground in liquid nitrogen and the total proteins were extracted using native extraction loading buffer (50 mM Tris-MES pH 8.0, 0.5 M Sucrose, 1 mM MgCl_2_, 10 mM EDTA, 5 mM DTT, and 1% w/v protease inhibitor cocktail S8830), 48 h after infiltration. Immunoblot analysis was performed as described previously [38, 39]. The antibodies used were anti-HA antibody (Abmart, 1: 2000) and goat anti-mouse HRP-conjugated antibody (1: 10, 000) [40].

### Virus-induced *TuRLK1* silencing

For silencing of *TuRLK1*, a 216-bp fragment of the gene was inserted in forward orientation into the Barley stripe mosaic virus RNAγ to form the recombinant vector BSMV: *TuRLK1*. The second fully expanded leaves of the PI428309 seedlings were infected with the recombinant vector, using BSMV: *GFP* (GFP, green fluorescent protein) as a control. The details of the assay were described in the previous publication [41]. Then, the fourth leaves were inoculated with *Pst CY33* and evaluated the resistance at 14 dpi. The expression levels of *TuRLK1* were determined by qRT-PCR [24].

### Histological analysis of *Pst CY33* growth

To visualize the substomatal vesicles (SSV), infection hyphae (IH), haustoria mother cells (HMCs) and so on of *Pst CY33*, the inoculated leaves were detached at 2, 3, 5 dpi and stained with WGA-FITC (L4895-10MG, Sigma) as described previously [15, 42]. The WGA-FITC-treated leaves were examined via blue light excitation, using a Zeiss LSM 880 confocal microscope.

### Single-cell transient gene expression assay

The single-cell transient expression assay, using biolistic particle delivery of plasmid DNA into plant epidermal cells, was performed as previous publication [43]. The reporter plasmid containing the β-glucuronidase (GUS) gene and the plasmid pUBI: *TuRLK1* were mixed before coating of the particles under the molar ratio of 1: 1, and the total DNA must be not more than 2.5 μg. Plasmid pUBI: *PGY* was used as control. The haustorium index was examined using microscopy by the mean of three independent experiments, observing at least 40 interactions in each repeat [24]. Single-cell transient gene expression method could also be used in subcellular localization assay.

### Agrobacterium-mediated transformation of Arabidopsis thaliana

The 1980-bp coding domain sequence of *TuRLK1* was cloned into binary vector pYBA 1143. The construct was introduced into the *Agrobacterium tumefaciens* strain GV3101. GV3101 with recombinant plasmid was suspended in a 5% (w/v) sucrose solution buffer containing 0.02% (v/v) Silwet L-77 and introduced the construct into *Arabidopsis thaliana* accession Columbia-0 (Col-0) through the floral dip method. The plants were cultured in a growth room under the conditions for seed setting. Positive individuals were screened on ½ Murashige and Skoog medium (½ MS) containing 0.005% (w/v) kanamycin [44, 45]. The stable transformants were identified in the T_3_ generation, which were used for further analysis.

### Primers

Primers used in this study are list in Additional file 2.

## Supplementary Information

**Additional file 1. **Multiple sequence alignment with TuRLK1 and 4 homologous proteins of TuRLK1 from different Triticeae accessions. TraesCS2A02G368700.2, from Chinese Spring, Triticum aestivum; TRITD2Av1G220310.4, from Triticum durum; TRIDC2AG053330.3 from Triticum turgidum and TraesFLD2A01G409100.1 from Fielder, Triticum aestivum. The identical residues are labeled in blue, whereas the less conserved residues are labeled in yellow.

**Additional file 2.** List of primers used in this study.

**Additional file 3.** Original image for Figure 3b.

## Data Availability

Sequence data of proteins (Fig. 1c) in this study can be found at http://202.194.139.32/ and https://www.arabidopsis.org/browse/Cereon/index.jsp.

## Abbreviations

NLR: Nucleotide-binding site (NBS) and leucine-rich repeat (LRR) domain receptor
PTI: Pathogen-/damage-derived molecules triggered immunity
*Bgt*: *Blumeria graminis* F. sp. *Tritici*
*Pst*: *Puccinia striiformis* F. sp. *Tritici*
VIGS: Barley stripe mosaic virus (BSMV)-induced gene silencing
ETI: Effector-triggered immunity
RLK: Receptor-like kinase
WGA: Wheat germ agglutinin
HB: Hyphal branches
HMC: Haustorial mother cells
H: Haustoria
IH: Infection hyphae
HR: Hypersensitive cell death response
ROS: Oxygen species
MAPKs: Mitogen-activated protein kinases
FPKM: Fragments Per Kilobase of exon model per Million mapped fragments

## References

[1] Ngou BPM, Jones JDG, Ding P (2022). Plant immune networks. Trends Plant Sci.

[2] Ngou BPM, Ding P, Jones JD (2022). Thirty years of resistance: Zig-zag through the plant immune system. Plant Cell.

[3] Wang W, Feng B, Zhou JM, Tang D (2020). Plant immune signaling: advancing on two frontiers. J Integr Plant Biol.

[4] Yuan M, Ngou BPM, Ding P, Xin X-F (2021). PTI-ETI crosstalk: an integrative view of plant immunity. Curr Opin Plant Biol.

[5] Tian H, Wu Z, Chen S, Ao K, Huang W, Yaghmaiean H, Sun T, Xu F, Zhang Y, Wang S (2021). Activation of TIR signalling boosts pattern-triggered immunity. Nature.

[6] Pruitt RN, Locci F, Wanke F, Zhang L, Saile SC, Joe A, Karelina D, Hua C, Frohlich K, Wan WL (2021). The EDS1-PAD4-ADR1 node mediates Arabidopsis pattern-triggered immunity. Nature.

[7] Ngou BPM, Ahn HK, Ding P, Jones JDG (2021). Mutual potentiation of plant immunity by cell-surface and intracellular receptors. Nature.

[8] Yuan M, Jiang Z, Bi G, Nomura K, Liu M, Wang Y, Cai B, Zhou JM, He SY, Xin XF (2021). Pattern-recognition receptors are required for NLR-mediated plant immunity. Nature.

[9] Tang D, Wang G, Zhou J-M (2017). Receptor Kinases in Plant-Pathogen interactions: more than pattern recognition. Plant Cell.

[10] Escocard de AzevedoManhaes AM, Ortiz-Morea FA, He P, Shan L (2021). Plant plasma membrane-resident receptors: Surveillance for infections and coordination for growth and development. J Integr Plant Biol.

[11] Beddow JM, Pardey PG, Chai Y, Hurley TM, Kriticos DJ, Braun HJ, Park RF, Cuddy WS, Yonow T (2015). Research investment implications of shifts in the global geography of wheat stripe rust. Nat Plants.

[12] Chen WQ, Wellings XM, Kang ZS, Liu TG (2014). Wheat stripe (yellow) rust caused by Puccinia striiformis f sp. Tritici.. Mol Plant Pathol..

[13] Chai Y, Pardey PG, Hurley TM, Senay SD, Beddow JM (2020). A probabilistic bio-economic assessment of the global consequences of wheat leaf rust. Phytopathology.

[14] Pakeerathan K, Bariana H, Qureshi N, Wong D, Hayden M, Bansal U (2019). Identification of a new source of stripe rust resistance Yr82 in wheat. Theor Appl Genet.

[15] Wang H, Zou S, Li Y, Lin F, Tang D (2020). An ankyrin-repeat and WRKY-domain-containing immune receptor confers stripe rust resistance in wheat. Nat Commun.

[16] Fu D, Uauy C, Distelfeld A, Blechl A, Epstein L, Chen X, Sela H, Fahima T, Dubcovsky J (2009). A kinase-START gene confers temperature-dependent resistance to wheat stripe rust. Science.

[17] Krattinger SG, Lagudah ES, Spielmeyer W, Singh RP, Huerta-Espino J, McFadden H, Bossolini E, Selter LL, Keller B (2009). A putative ABC transporter confers durable resistance to multiple fungal pathogens in wheat. Science.

[18] Moore JW, Herrera-Foessel S, Lan C, Schnippenkoetter W, Ayliffe M, Huerta-Espino J, Lillemo M, Viccars L, Milne R, Periyannan S (2015). A recently evolved hexose transporter variant confers resistance to multiple pathogens in wheat. Nat Genet.

[19] Marchal C, Zhang J, Zhang P, Fenwick P, Steuernagel B, Adamski NM, Boyd L, McIntosh R, Wulff BBH, Berry S (2018). BED-domain-containing immune receptors confer diverse resistance spectra to yellow rust. Nat Plants.

[20] Klymiuk V, Yaniv E, Huang L, Raats D, Fatiukha A, Chen S, Feng L, Frenkel Z, Krugman T, Lidzbarsky G (2018). Cloning of the wheat Yr15 resistance gene sheds light on the plant tandem kinase-pseudokinase family. Nat Commun.

[21] Zhang C, Huang L, Zhang H, Hao Q, Lyu B, Wang M, Epstein L, Liu M, Kou C, Qi J (2019). An ancestral NB-LRR with duplicated 3’UTRs confers stripe rust resistance in wheat and barley. Nat Commun.

[22] Bouvet L, Holdgate S, James L, Thomas J, Mackay IJ, Cockram J (2022). The evolving battle between yellow rust and wheat: implications for global food security. Theor Appl Genet..

[23] Xu Y, Zou S, Zeng H, Wang W, Wang B, Wang H, Tang D (2022). A NAC Transcription Factor TuNAC69 Contributes to ANK-NLR-WRKY NLR-Mediated Stripe Rust Resistance in the Diploid Wheat Triticum urartu. Int J Mol Sci.

[24] Zou SH, Wang H, Li YW, Kong ZS, Tang DZ (2018). The NB-LRR gene Pm60 confers powdery mildew resistance in wheat. New Phytol.

[25] Couto D, Zipfel C (2016). Regulation of pattern recognition receptor signalling in plants. Nat Rev Immunol.

[26] Gao C, Sun P, Wang W, Tang D (2021). Arabidopsis E3 ligase KEG associates with and ubiquitinates MKK4 and MKK5 to regulate plant immunity. J Integr Plant Biol.

[27] Liu N, Hake K, Wang W, Zhao T, Romeis T, Tang D (2017). CALCIUM-DEPENDENT PROTEIN KINASE5 associates with the truncated NLR protein TIR-NBS2 to contribute to exo70B1-mediated immunity. Plant Cell.

[28] Adam L, Somerville SC (1996). Genetic characterization of five powdery mildew disease resistance loci in Arabidopsis thaliana. Plant J.

[29] Liu S, Huang S, Zeng Q, Wang X, Yu R, Wang Q, Singh RP, Bhavani S, Kang Z, Wu J (2021). Refined mapping of stripe rust resistance gene YrP10090 within a desirable haplotype for wheat improvement on chromosome 6A. Theor Appl Genet.

[30] Wang X, Zhai T, Zhang X, Tang C, Zhuang R, Zhao H, Xu Q, Cheng Y, Wang J, Duplessis S (2021). Two stripe rust effectors impair wheat resistance by suppressing import of host Fe-S protein into chloroplasts. Plant Physiol.

[31] Zhang Y, Bai Y, Wu G, Zou S, Chen Y, Gao C, Tang D (2017). Simultaneous modification of three homoeologs of TaEDR1 by genome editing enhances powdery mildew resistance in wheat. Plant J.

[32] Yang D, Li S, Xiao Y, Lu L, Zheng Z, Tang D, Cui H (2021). Transcriptome analysis of rice response to blast fungus identified core genes involved in immunity. Plant Cell Environ.

[33] Yang H (2016). Xu X-l, Ma H-m, Jiang J: Integrative analysis of transcriptomics and proteomics of skeletal muscles of the Chinese indigenous Shaziling pig compared with the Yorkshire breed. BMC Genet.

[34] Zhao C, Waalwijk C, de Wit PJGM, Tang D, van der Lee T (2013). RNA-Seq analysis reveals new gene models and alternative splicing in the fungal pathogen Fusarium graminearum. BMC Genomics.

[35] Nie H, Zhao C, Wu G, Wu Y, Chen Y, Tang D (2012). SR1, a calmodulin-binding transcription factor, modulates plant defense and ethylene-induced senescence by directly regulating NDR1 and EIN3. Plant Physiol.

[36] Koch E, Slusarenko A (1990). Arabidopsis is susceptible to infection by a downy mildew fungus. Plant Cell.

[37] Liu L, Zhang Y, Tang S, Zhao Q, Zhang Z, Zhang H, Dong L, Guo H, Xie Q (2010). An efficient system to detect protein ubiquitination by agroinfiltration in Nicotiana benthamiana. Plant J.

[38] Periyannan S, Moore J, Ayliffe M, Bansal U, Wang X, Huang L, Deal K, Luo M, Kong X, Bariana H (2013). The gene Sr33, an ortholog of Barley Mla Genes, encodes resistance to wheat stem rust race Ug99. Science.

[39] Zhao C, Nie H, Shen Q, Zhang S, Lukowitz W, Tang D (2014). EDR1 Physically Interacts with MKK4/MKK5 and Negatively Regulates a MAP Kinase Cascade to Modulate Plant Innate Immunity. PLoS Genet.

[40] Zhao Y, Wu G, Shi H, Tang D (2019). RECEPTOR-LIKE KINASE 902 Associates with and Phosphorylates Brassinosteroid-Signaling Kinase1 to regulate plant immunity. Mol Plant.

[41] Holzberg S, Brosio P, Gross C, Pogue GP (2002). Barley stripe mosaic virus-induced gene silencing in a monocot plant. Plant J.

[42] Zhang W, Chen S, Abate Z, Nirmala J, Rouse MN, Dubcovsky J (2017). Identification and characterization of Sr13, a tetraploid wheat gene that confers resistance to the Ug99 stem rust race group. Proc Natl Acad Sci U S A.

[43] Shen Q-H, Saijo Y, Mauch S, Biskup C, Bieri S, Keller B, Seki H, Ülker B, Somssich IE, Schulze-Lefert P (2007). Nuclear activity of MLA immune receptors links isolate-specific and basal disease-resistance responses. Science.

[44] Wu G, Liu S, Zhao Y, Wang W, Kong Z, Tang D (2015). Enhanced disease resistance4 associates with clathrin Heavy Chain2 and modulates plant immunity by regulating relocation Of Edr1 In Arabidopsis. Plant Cell.

[45] Clough SJ, Bent AF (1998). Floral dip: a simplified method for Agrobacterium-mediated transformation of Arabidopsis thaliana. Plant J.

