# Supplementary material for: TuRLK1, a leucine-rich repeat receptor-like kinase, is indispensable for stripe rust resistance of YrU1 and confers broad resistance to multiple pathogens
Source: BMC Plant Biol. 2022 Jun 8;22:280. doi: 10.1186/s12870-022-03679-6 (PMC9175386; doi:10.1186/s12870-022-03679-6)
Supplement: Supplementary file 1 — Additional file 1. Multiple sequence alignment with TuRLK1 and 4 homologous proteins of TuRLK1 from different Triticeae accessions. TraesCS2A02G368700.2, from Chinese Spring, Triticum aestivum; TRITD2Av1G220310.4, from Triticum durum; TRIDC2AG053330.3 from Triticum turgidum and TraesFLD2A01G409100.1 from Fielder, Triticum aestivum. The identical residues are labeled in blue, whereas the less conserved residues are labeled in yellow. [file 12870_2022_3679_MOESM1_ESM.pdf]

Sequence	Alignment	
TuRLK1 PI428309 *Triticum urartu*	MARLLLGVSLLAMALGLGCCASIAPAPEPSDSASEPSVSDDVRALLAFKRAIDD.PRAELSNWNTSEPDHCWWSGVWCSLSDGRVVALELSNSSLSGFLAPEIGSLTSLQKLILDHNAFT	119
TraesCS2A02G368700.2 Chinese Spring *Triticum aestivum*	MARLLLGVSLLAMALGLGCCASIAPAPEPSDSASEPSVSDDVRALLAFKRAIDDDPRAELSNWNTSEPDHCWWSGVWCSLSDGRVVALELSNSSLSGFLAPEIGSLTSLQKLILDHNAFT	120
TRITD2Av1G220310.4 *Triticum durum*	MARLLLGVSLLAMALGLGCCASIAPAPEPSDSASEPSVSDDVRALLAFKRAIDDDPRAELSNWNTSEPDHCWWSGVWCSLSDGRVVALELSNSSLSGFLAPEIGSLTSLQKLILDHNAFT	120
TRIDC2AG053330.3 *Triticum turgidum*	MARLLLGVSLLAMALGLGCCASIDPAPEPSDSASEPSVSDDVRALLAFKRAIDDDPRAELSSWNTSEPDHCWWSGVWCSLSDGRVVALELSNSSLSGFLAPEIGSLTSLQKLILDHNAFT	120
TraesFLD2A01G409100.1 Fielder *Triticum aestivum*	MARLLLGVSLLAMALGLGCCASIAPAPEPSDSASEPSVSDDVRALLAFKRAIDDDPRAELSNWNTSEPDHCWWSGVWCSLSDGRVVALELSNSSLSGFLAPEIGSLTSLQKLILDHNAFT	120
Consensus	marlllgvsllamalglgccasi papepsdsasepsvsddvrallafkraidd praels wntsepdhcwwsgvwcslsdgrvvalelsnsslsgflapeigsltslqklildhnaft	

Sequence	Alignment	
TuRLK1 PI428309 *Triticum urartu*	GSIPREIGKLKNLTVLNLSTNQLEGPIPSEAGDMQNITTIDLHANRLSGAIPPELGNLTNLKELRLSNNSLTGTIPGSNDSIVVSTKKEDQVGLCQLAQLTDIDLSNNLLAGSIPACLGH	239
TraesCS2A02G368700.2 Chinese Spring *Triticum aestivum*	GSIPREIGKLKNLTVLNLSTNQLEGPIPSEAGDMQNITTIDLHANRLSGAIPPELGKLANLKELRLSNNSLTGTIPGSNDSIMVSTKKEDQVGLCQLAQLTDIDLSNNLLAGSIPACLGH	240
TRITD2Av1G220310.4 *Triticum durum*	GSIPREIGKLKNLTVLNLSTNQLEGPIPSEAGDMQNITTIDLHANRLSGAIPPELGKLANLKELRLSNNSLTGTIPGSNDSIMVSTKKEDQVGLCQLAQLTDIDLSNNLLAGSIPACLGH	240
TRIDC2AG053330.3 *Triticum turgidum*	GSIPREIGKLKNLTVLNLSTNQLEGPIPSEAGDMQNITTIDLHANRLSGAIPPELGKLANLKELRLSNNSLTGTIPGSNDSIMVSTKKEDQVGLCQLAQLTDIDLSNNLLAGSIPACLGH	240
TraesFLD2A01G409100.1 Fielder *Triticum aestivum*	GSIPREIGKLKNLTVLNLSTNQLEGPIPSEAGDMQNITTIDLHANRLSGAIPPELGKLANLKELRLSNNSLTGTIPGSNDSIMVSTKKEDQVGLCQLAQLTDIDLSNNLLAGSIPACLGH	240
Consensus	gsipreigklknltvlnlstnqlegpipseagdmqnittidlhanrlsgaippelg l nlkelrlsnnsltgtipgsndsi vstkkedqvglcqlaqltdidlsnnllagsipaclgh	

Sequence	Alignment	
TuRLK1 PI428309 *Triticum urartu*	IQRSSMVGNCFHNNDTRNRPDWECGNSMDAGKDNNNTSIGEDGQRGRVIQPLWLLIVEVVTGVSVLSILTLCAIAGLRRRKDRSSRRGVPWTRALSWKENNVISIDDDLLGNVPKISRQE	359
TraesCS2A02G368700.2 Chinese Spring *Triticum aestivum*	IQRSSMVGNCFHNNDTRNRPDWECGNSMDAGKDNNNTSIGEDGQRGRVIQPLWLLIVEVVTGVSVLSILTLCAIAGLRRRKDRSSRRGVPWTRALSWKENNVISIDDDLLGNVPKISRQE	360
TRITD2Av1G220310.4 *Triticum durum*	IQRSSMVGNCFHNNDTRNRPDWECGNSMDAGKDNNNTSIGEDGQRGRVIQPLWLLIVEVVTGVSVLSILTLCAIAGLRRRKDRSSRRGVPWTRALSWKENNVISIDDDLLGNVPKISRQE	360
TRIDC2AG053330.3 *Triticum turgidum*	IQRSSMVGNCFHNNDTRNRPDWECGNSMDAGKDNNNTSIGEDGQRGRVIQPLWLLIVEVVTGVSVLSILTLCAIAGLRRRKDRSSRRGVPWTRALSWKENNVISIDDDLLGNVPKISRQE	360
TraesFLD2A01G409100.1 Fielder *Triticum aestivum*	IQRSSMVGNCFHNNDTRNRPDWEC.......KDNNNTSIGEDGQRGRVIQPLWLLIVEVVTGVSVLSILTLCAIAGLRRRKDRSSRRGVPWTRALSWKENNVISIDDDLLGNVPKISRQE	353
Consensus	iqrssmvgncfhnndtrnrpdwec kdnnntsigedgqrgrviqplwllivevvtgvsvlsiltlcaiaglrrrkdrssrrgvpwtralswkennvisidddllgnvpkisrqe	

Sequence	Alignment	
TuRLK1 PI428309 *Triticum urartu*	LAEACEDFSNIIGSSQETVVYKGTMKDGREIAVVSMSASVHYWTNYVELYFQKEVVEVARLSHENAGKMVGYCKSSDPFSRMVVFEYPSNGTLYEHLHDVEGCQLSWPRRMKIALSIARV	479
TraesCS2A02G368700.2 Chinese Spring *Triticum aestivum*	LAEACEDFSNIIGSSQETVVYKGTMKDGREIAVVSMSASVHHWTNYVELYFQKEVVEVARLSHENAGKMVGYCKSSDPFSRMVVFEYPSNGTLYEHLHDVEGCQLSWPRRMKIALSIARV	480
TRITD2Av1G220310.4 *Triticum durum*	LAEACEDFSNIIGSSQETVVYKGTMKDGREIAVVSMSASVHHWTNYVELYFQKEVVEVARLSHENAGKMVGYCKSSDPFSRMVVFEYPSNGTLYEHLHDVEGCQLSWPRRMKIALSIARV	480
TRIDC2AG053330.3 *Triticum turgidum*	LAEACEDFSNIIGSSQETVVYKGTMKDGREIAVVSMSASVHHWTNYVELYFQKEVVEVARLSHENAGKMVGYCKSSDPFSRMVVFEYPSNGTLYEHLHDVEGCQLSWPRRMKIALSIARV	480
TraesFLD2A01G409100.1 Fielder *Triticum aestivum*	LAEACEDFSNIIGSSQETVVYKGTMKDGREIAVVSMSASVHHWTNYVELYFQKEVVEVARLSHENAGKMVGYCKSSDPFSRMVVFEYPSNGTLYEHLHDVEGCQLSWPRRMKIALSIARV	473
Consensus	laeacedfsniigssqetvvykgtmkdgreiavvsmsasvh wtnyvelyfqkevvevarlshenagkmvgyckssdpfsrmvvfeypsngtlyehlhdvegcqlswprrmkialsiarv	

Sequence	Alignment	
TuRLK1 PI428309 *Triticum urartu*	LRHLHTELQPPFAVAALASSSVYLTEDFSPKIIDFERWRGLVGKPLLSSGCVVNGGGGHSNGVVDSRHVRFMDVQANTFAFGVILLELISGRASLSKDTDDLVNWARKHLEQAGEFGKLV	599
TraesCS2A02G368700.2 Chinese Spring *Triticum aestivum*	LRHLHTELQPPFAVAALASSSVYLTEDFSPKIIDFERWRGLVGKPLLSSGCVVNGGGGHSNGVVDSRHVRFMDVQANTFAFGVILLELISGRASLSKDTDDLVNWARKHLEQAGEFGKLV	600
TRITD2Av1G220310.4 *Triticum durum*	LRHLHTELQPPFAVAALASSSVYLTEDFSPKIIDFERWRGLVGKPLLSSGCVVNGGGGHSNGVVDSRHVRFMDVQANTFAFGVILLELISGRASLSKDTDDLVNWARKHLEQAGEFGKLV	600
TRIDC2AG053330.3 *Triticum turgidum*	LRHLHTELQPPFAVAALASSSVYLTEDFSPKIIDFERWRGLVGKPLLSSGCVVNGGGGHSNGVVDSRHVRFMDVQANTFAFGVILLELISGRASLSKDTDDLVNWARKHLEQAGEFGKLV	600
TraesFLD2A01G409100.1 Fielder *Triticum aestivum*	LRHLHTELQPPFAVAALASSSVYLTEDFSPKIIDFERWRGLVGKPLLSSGCVVNGGGGHSNGVVDSRHVRFMDVQANTFAFGVILLELISGRASLSKDTDDLVNWARKHLEQAGEFGKLV	593
Consensus	lrhlhtelqppfavaalasssvyltedfspkiidferwrglvgkpllssgcvvnggggghsngvvdsrhvrfmdvqantfafgvillelisgraslskdtddlvnwarkhleqagefgklv	

Sequence	Alignment	
TuRLK1 PI428309 *Triticum urartu*	DPKLRSVGQESLGIICNVVNLCIDAEPSRRPSMNMIGAILEEGVDTSVRDSSLAWAEAVIS	660
TraesCS2A02G368700.2 Chinese Spring *Triticum aestivum*	DPKLRSVGQESLGIICNVVNLCIDAEPSRRPSMNMIGAILEEGVDTSVRDSSLAWAEAAIS	661
TRITD2Av1G220310.4 *Triticum durum*	DPKLRSVGQESLGIICNVVNLCIDAEPSRRPSMNMIGAILEEGVDTSVRDSSLAWAEAAIS	661
TRIDC2AG053330.3 *Triticum turgidum*	DPKLRSVGQESLGIICNVVNLCIDAEPSRRPSMNMIGAILEEGVDTS..............	647
TraesFLD2A01G409100.1 Fielder *Triticum aestivum*	DPKLRSVGQESLGIICNVVNLCIDAEPSRRPSMNMIGAILEEGVDTSVRDSSLAWAEAAIS	654
Consensus	dpklrsvgqeslgiicnvvnlcidaepsrrpsmnmigaileegvdts	
