# Supplementary material for: TuRLK1, a leucine-rich repeat receptor-like kinase, is indispensable for stripe rust resistance of YrU1 and confers broad resistance to multiple pathogens
Source: BMC Plant Biol. 2022 Jun 8;22:280. doi: 10.1186/s12870-022-03679-6 (PMC9175386; doi:10.1186/s12870-022-03679-6)
Supplement: Supplementary file 2 — Additional file 2. List of primers used in this study. [file 12870_2022_3679_MOESM2_ESM.docx]

| **Primer name** | **Sequence** |
| --- | --- |
| **For cloning** |  |
| *TuRLK1*-F | cgcacggggctctagagatgacta |
| *TuRLK1*-R | agcagcgaagcaaacaagggtca |
| BP-*TuRLK1* (N-tag)-F | ggggacaagtttgtacaaaaaagcaggcttcatggcgaggctgctgctcggg |
| BP-*TuRLK1* (N-tag)-R | ggggaccactttgtacaagaaagctgggtcctaggagatgaccgcctccgcccac |
| BP-*TuRLK1* (C-tag)-F | ggggacaagtttgtacaaaaaagcaggcttcatggcgaggctgctgctcggggtc |
| BP-*TuRLK1* (C-tag)-R | ggggaccactttgtacaagaaagctgggtggagatgaccgcctccgcccactag |
| **For qRT-PCR analysis** |  |
| *TuRLK1-*qRT*-*F | ctcagacgacgtgcgtgcg |
| *TuRLK1*-qRT-R | aggatccaatctctggtgcgag |
| *ACTIN*-F | gcacctgaacctttctgaaccaa |
| *ACTIN*-R | tcgcttacgtggcccttgat |
| **For VIGS** |  |
| RNAγ.*TuRLK1*-NheI F | ttttttttttttttagctagcggtggtagaagtggccagattg |
| RNAγ.*TuRLK1*-NheI R | gattcttcttccgttgctagcgtgtgcaggtgtctgagcacg |
| **For localization** |  |
| 35S-TuRLK1-GFP-F | ctagaggatccggtacccgggatggcgaggctgctgctcggg |
| 35S-TuRLK1-GFP-R | cgcccttgctcaccatcccgggggagatgaccgcctccgcc |

**Additional file 1** List of primers used in this study
