# Supplementary figures and images for: TuRLK1, a leucine-rich repeat receptor-like kinase, is indispensable for stripe rust resistance of YrU1 and confers broad resistance to multiple pathogens
Source: BMC Plant Biol. 2022 Jun 8;22:280. doi: 10.1186/s12870-022-03679-6 (PMC9175386; doi:10.1186/s12870-022-03679-6)

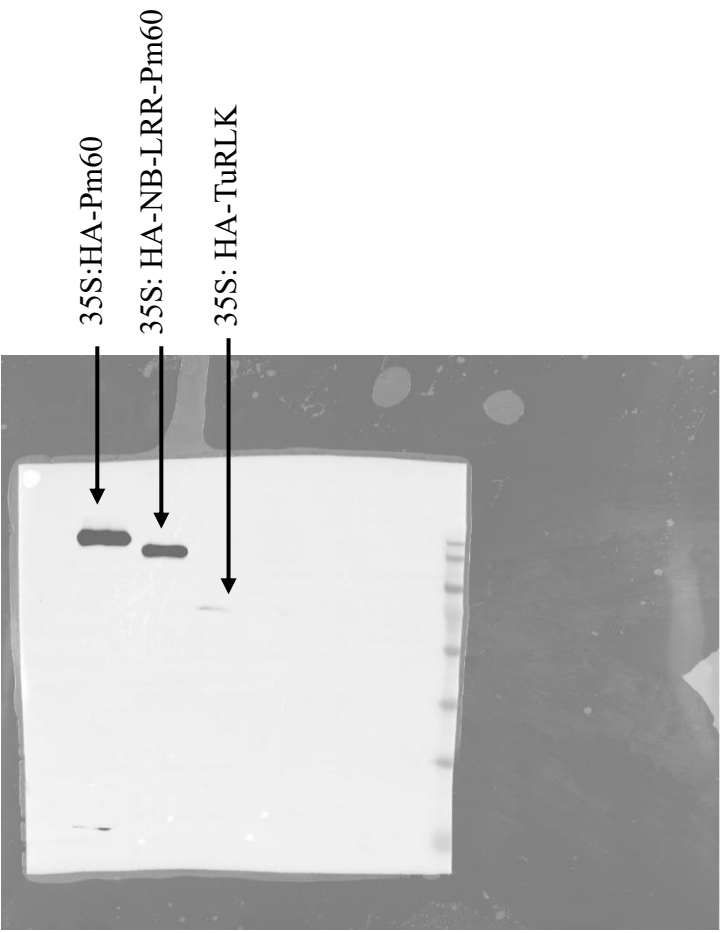

Supplement: Supplementary file 3 — Additional file 3. Original image for Figure 3b. [file 12870_2022_3679_MOESM3_ESM.pdf]
